# Estrogen receptor subtype agonist activation in human cutaneous squamous cell carcinoma cells modulates expression of CD55 and Cyclin D1

**DOI:** 10.17179/excli2019-1541

**Published:** 2019-08-08

**Authors:** Jing Lan, Xing-Hua Gao, Rashmi Kaul

**Affiliations:** 1 Department of Dermatology, the First Affiliated Hospital of China Medical University, Shenyang, Liaoning, 110001, China; 2 Department of Biochemistry and Microbiology, Oklahoma State University, Center for Health Sciences, Tulsa, Oklahoma, 74107, United States

**Keywords:** estrogen, estrogen receptor, cutaneous squamous cell carcinoma, Cyclin D1, CD55, GPR30

## Abstract

Clinical studies indicate gender bias in cutaneous squamous cell carcinoma (cSCC) incidence with worse prognosis observed in males than in females, suggesting estrogen-mediated protection. In contrast, recent clinical population studies show risk of cSCC by use of oral contraceptives, thus raising controversy. However, animal studies indicate a protective role of estrogen and estrogen receptor (ER)s in cSCC. Currently we have a poor understanding of ERs that are expressed in human cSCC cells and their possible role in malignant transformation. The focus of current study was to determine ER subtype specific expression on cSCC A431 cells and investigate if ER agonist based activation modulates tumor markers CD55 and Cyclin D1 in the cells. ERα, ERβ and G protein-coupled receptor (GPR30) subtype expression at mRNA and protein level was determined in human cSCC A431 cells by reverse transcription-quantitative polymerase chain reaction (RT-qPCR) and Western blotting, respectively. The localization of ER subtypes was determined by confocal microscopy. ER subtype agonist based activation on A431 cells was performed to investigate their role in modulating mRNA and protein expression of tumor markers CD55 and Cyclin D1. A431 cells differentially expressed all three ER subtypes- ERα, ERβ and GPR30 with GPR30 expression being the highest. Confocal studies confirmed that all three ER subtypes were expressed in the cytoplasm and ERα and ERβ lacked nuclear expression. Agonist based activation of both ERα and GPR30 significantly upregulated Cyclin D1 and CD55 expression. Blocking of GPR30 led to significantly downregulation of both Cyclin D1 and CD55 expression. In contrast to ERα and GPR30, ERβ activation significantly downregulated CD55 expression. Taken together, here we demonstrate for the first time that all three ERs- ERα, ERβ and GPR30 are expressed in human A431 cSCC cells and further ER agonist based activation modulates the expression of tumor markers CD55 and Cyclin D1.

## Introduction

Cutaneous squamous cell carcinoma (cSCC) is the second most common malignancy especially among Caucasians in the United States (Yesantharao et al., 2017[40]). Animal model studies have confirmed the gender differences and protective role of estrogen in development of cSCC (Thomas-Ahner et al., 2007[35]; Mancuso et al., 2009[22]). Further, in population studies, gender based data in the United States indicate that the lifetime risk of cSCC incidence is higher in men than women (Pollock, 2001[29]) and metastatic cSCC is more common in men (Burton et al., 2016[4]). Additionally, postmenopausal women show a higher risk of vulvar SCC than premenopausal women (Nugent et al., 2011[28]). Mouse model studies have further confirmed keratinocyte tumorogenesis due to estrogen deficiency (Mancuso et al., 2009[22]). However, a recent clinical epidemiological study had controversial findings about estrogen by reporting that oral contraceptive use is associated with a risk of cSCC incidence (Kuklinski et al., 2016[18]). Currently, detailed studies in populations are lacking to confirm the role of estrogen in cSCC. Hence, there is a huge gap in understanding of estrogen related pathogenesis in human cSCC development. Estrogen has been reported to be involved in normal cell physiology as well as malignancies (Yue et al., 2010[41]; Chen et al., 2018[6]; Ito et al., 2001[15]). In order to understand the underlying hormonal etiology, improve diagnosis and prognosis of cSCC, we urgently need detailed basic studies to investigate relationship between estrogen and development of cSCC.

Estrogen acts on cells via estrogen receptor (ER)s, we therefore studied the effects of ERs on cSCC to explore the impacts of estrogen on cSCC. ERs include ERα, ERβ and G protein-coupled receptor (GPR30). ERα and ERβ have been detected broadly in various cancers (Yue et al., 2010[41]; Chen et al., 2018[6]; Ito et al., 2001[15]) exerting different roles in regulating malignant progression. ERα and ERβ function as transcription factors for regulating the expression of various genes involved in inflammation, cell cycle, proliferation and apoptosis (Heldring et al., 2007[13]). An experimental cSCC mouse model study reported that the degree of malignancy in cSCC is associated with the ratio of ERα / ERβ (Logotheti et al., 2012[21]). Till date, we have poor understanding of ERα and ERβ on malignant progression in human cSCC. GPR30 is a seven transmembrane-domain G protein-coupled receptor which mediates non-genomic signaling of estrogen to regulate cell growth and has been discovered expressed in human breast, ovarian, bladder cancers (Tian et al., 2017[36]; Zhu et al., 2018[42]; Huang et al., 2015[14]) and cutaneous malignant melanoma (Sun et al., 2017[32]). However, studies related to expression of GPR30 and its activation in human cSCC are completely lacking in the literature.

Cellular proliferation predicts tumor malignancy behavior correlated with tumor growth and metastatic potential (van Diest et al., 1998[37]). Overexpression of proliferation marker Cyclin D1 is detected in many tumors and associated with malignant progression (Yang et al., 2002[39]) and poor prognosis (Ahlin et al., 2017[1]). Though the correlation between Cyclin D1 and ERs has been studied (Nakamura et al., 2013[26]; Guo et al., 2015[12]) in some cancer cells, the modulation on Cyclin D1 expression by ERs in human cSCC cells is yet to be explored.

There is established evidence in the literature that chronic inflammation increases the risk of cancer and promotes tumor progression (Multhoff et al., 2011[24]). As part of the innate immunity, complement regulatory protein (CRP) such as CD55 is often upregulated during inflammation (Kawano, 2000[16]) and cancer development (Murray et al., 2000[25]; Li et al., 2001[20]). Overexpressed CD55 leads to a poor outcome in certain cancers (Durrant et al., 2003[9]), additionally, human cSCC cells also show resistance to complement lysis due to the presence of CD55 (Whitlow and Klein, 1997[38]). Therefore, modulation of CD55 may be an important step for cell malignant progression in cSCC.

In the current study, we hypothesized that estrogen via ER activation in cSCC cells impacts cancer progression by modulating Cyclin D1 and CD55. Therefore, we investigated the expression of ER subtypes in human cSCC A431 cells and the effects of ER subtype activation by ER agonists on the expression of tumor markers CD55 and Cyclin D1.

## Materials and Methods

### Chemicals

ERα agonist 4,4′,4″-(4-propyl [1H] pyrazole-1,3,5-triyl)-trisphenol (PPT), ERβ agonist 3-bis(4-hydroxyphenyl)-propionitrile (DPN), GPR30 agonist G1, GPR30 antagonist G15 and 17β-estradiol (E2) were purchased from Cayman Chemicals (Ann Arbor, United States). PPT and DPN drugs were dissolved in 100 % ethanol; G1, G15 and E2 drugs were dissolved in 100 % DMSO. These drugs from the stock solution were then diluted to required concentrations in cell culture medium.

### Cell line and cell culture

Human cSCC cell line A431 was obtained from Dr. Santosh Katiyar (University of Alabama at Brimingham, United States). The cells were grown in phenol red free DMEM (Thermo Fisher Scientific, Waltham, United States) supplemented with 10 % fetal bovine serum (FBS, Atlanta Biologicals Inc, Flowery Branch, Georgia), 1 % sodium pyruvate and 1 % penicillin-streptomycin-glutamine (Thermo Fisher Scientific, Waltham, United States). Cells were maintained in 5 % CO_2_ incubator at 37 °C.

### Cell viability assay

Cell viability of the cells was evaluated by MTT (3- (4, 5-Dimethylthiazol-2-yl)- 2,5-diphenyltetrazolium bromide (Thermo Fisher Scientific, Waltham, USA) assay. A431 cells were seeded in 96-well tissue culture plates at a density of 1x10^4^/well and allowed to attach overnight. The next day, the cells were starved with serum deprived medium for 20 hours and then treated with vehicle (0.01 % of DMSO or ethanol), PPT, DPN, G1 or E2+G15 for 24 hours. 10 µM of MTT (0.5 mg/ml in sterilized 1x Dulbecco's Phosphate-Buffered Saline) was then added into each well and incubated for 2 hours at 37 °C. Then the formazan crystal was dissolved in 100 µl DMSO added in each well. After 5 minutes mixing, the absorbance of dissolved product was measured using SpectraMax Plus 384 Microplate Reader (Molecular Devices, California, United States) at a wavelength of 540 nm. Percentage of cell viability was calculated based on vehicle treated cells serving as negative control.

### RNA isolation and reverse transcription- quantitative polymerase chain reaction (RT-qPCR) analysis

A431 cells were seeded in 6-well tissue culture plates at a density of 1x10^5^/well and allowed to attach overnight. The next day, the cells were starved with serum deprived medium for 20 hours and then treated with vehicle (0.01 % of DMSO or ethanol), PPT, DPN, G1 or E2+G15 for 24 hours. Total RNA was extracted from vehicle treated and chemicals treated cells using TRIzol reagent (Thermo Fisher Scientific, Waltham, United States). The quality and concentration of RNA was assessed using NanoDrop 1000 Spectrophotometer (Thermo Fisher Scientific, Waltham, United States). cDNA was prepared using QuantiNova reverse transcription kit (Qiagen, Hilden, Germany) according to the manufacturer's instructions. Real-time quantitative PCR reaction mixture was carried out using ERα (ESR1), ERβ (ESR2), GPR30, Cyclin D1 (CCND1) and CD55 gene with RPL 13A gene used as an internal control (primers sequences are shown in Table 1(Tab. 1)) and Powerup Syber Green master mix (Applied Biosystems Inc, Foster City, California, United States). The reaction analysis was performed on StepOne Real-Time PCR system (Applied Biosystems, Foster City, California, United States). The data was calculated using the 2^−^ΔΔCt method and expressed as fold change relative to vehicle treated cells. 

### Western blotting assay

A431 cells were seeded in 6-well tissue culture plates at a density of 1x10^5^/well and allowed to attach overnight. The next day, the cells were starved with serum deprived medium for 20 hours and then treated with vehicle (0.01 % of DMSO or ethanol), PPT, DPN, G1 or E2+G15 for 24 hours. After washing, the cells were lysed with RIPA buffer (20 mM Tris, 150 mM NaCl, 1 % NP40, 0.5 % sodium deoxycholate, 0.1 % SDS, pH 7.4) containing protease and phosphatase inhibitor cocktail and phenylmethanesulfonyl fluoride (Thermo Fisher Scientific; Waltham, MA/United States). Total protein concentrations of the cell extracts were determined using Pierce BCA protein assay kit (Thermo Fisher Scientific, Waltham, United States). Protein samples were denatured by heating at 99 °C for 5 minutes. Proteins were separated on 4-12 % Bis-Tris protein gel (Thermo Fisher Scientific, Waltham, MA/United States) and transferred to nitrocellulose blotting membrane. After blocking non-specific staining with 5 % milk in TBST for 1 hour at room temperature, the membranes were incubated with primary antibodies specific for human ERα (1:500, rabbit monoclonal against human ERα, Abcam, Cambridge, MA/United States), human ERβ (1:1000, rabbit polyclonal against human ERβ, Abcam, Cambridge, MA/United States), human GPR30 (1:250, rabbit polyclonal against human GPR30, Abcam, Cambridge, MA/United States), human CD55 (1:5000, rabbit monoclonal anti-human CD55, Abcam, Cambridge, MA/United States) or human Cyclin D1 (1:5000, rabbit monoclonal against human Cyclin D1, Abcam, Cambridge, MA/United States) in 5 % milk in TBST at 4 °C overnight. Human β-actin (1:3000, rabbit polyclonal against human β-actin, Abcam, Cambridge, MA/United States) or human β-tubulin (1:500, rabbit polyclonal against humanβ-tubulin, Santa Cruz Biotechnology, Dallas, TX/United States) primary antibody was used as internal control. After additional washes the next day, the membranes were incubated with alkaline phosphatase conjugated goat anti-rabbit secondary antibody (1:3000, Cell Signaling, Danvers, MA/United States) for 1 hour at room temperature. The membranes were then developed using ECF substrate (Careforde Safaty & Scientific, Chicago, IL/United States) and scanned on Typhoon 9410 Variable Mode Imager (GE Healthcare Life Science, Chicago, IL/United States).Protein bands were analyzed using image J software (NIH, Bethesda, MD/United States).

### Confocal microscopy

A431 cells were seeded in 8-well chamber slide at a density of 4x10^4^/well and cultured for 48 hours. The cells were then washed with PBS and fixed with 4 % paraformaldehyde (EMD, Burlington, MA/United States) in PBS and incubated at room temperature for 1 hour. After washing, the fixed cells were permeabilized with 0.1 % Triton X-100 in PBS for 10 minutes at room temperature. Non-specific binding was blocked with 3 % bovine serum albumin (BSA, EMD, Burlington, MA/United States) in PBS for 30 minutes. The cells were then incubated with primary antibodies specific for human ERα (1:150, rabbit monoclonal against human ERα, Abcam, Cambridge, MA/United States), human ERβ (1:2000, rabbit polyclonal against human ERβ, Abcam, Cambridge, MA/United States) or human GPR30 (1:400, rabbit polyclonal against human GPR30, Abcam, Cambridge, MA/United States) in 3 % BSA in PBS at 4˚C overnight. After washing steps, the cells were incubated with Alexa fluor 488 goat anti-rabbit IgG (1:200, Abcam, Cambridge, MA/United States) supplemented with phalloidin (1:100, Abcam, Cambridge, MA/United States) in 3 % BSA in PBS at room temperature for 1 hour. The cells were then washed and counterstained with DAPI provided in mounting medium (Santa Cruz Biotechnology, Dallas, TX/United States). Negative controls were included where primary or secondary antibody was omitted. The stained cells were observed by Leica TCS SPE confocal microscope with a 63x oil-immersion objective lens. Z-stack analysis was performed for all images. Relative fluorescence intensity was estimated using NIS-Elements imaging software.

### Statistical analysis

All experiments were repeated at least 3 times. Data are presented as mean ± SEM. Statistical analysis was performed using the Graphpad Prism software 6.02 (Graphpad Software, Inc. California, United States). Significant differences were determined by unpaired t test or one-way ANOVA followed by Dunnett's or Tukey's multiple comparisons test. A P value of < 0.05 was considered as statistically significant.

## Results

### A431 cells have differential expression of ERα, ERβ and GPR30

**Figure 1:**
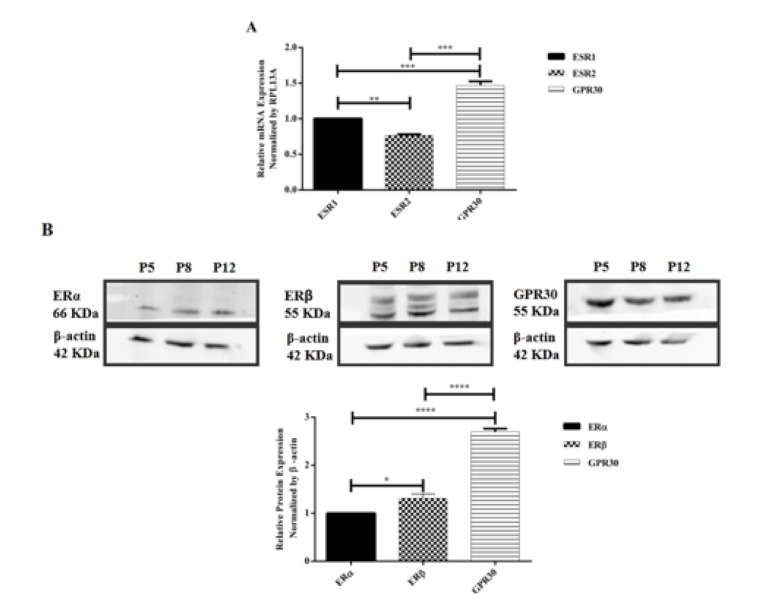
Basal expression of ER subtypes in A431 cells. (A) Expression of ESR1, ESR2 and GPR30 mRNA in A431 cells was determined by RT-qPCR. RPL13A was used as an internal control. The relative expression of ESR2 and GPR30 mRNA was normalized to ESR1. (B) Expression of ERα, ERβ and GPR30 protein in A431 whole lysate (passage 5, 8 and 12) was assessed using Western blotting assay. β-actin was used as an internal control. The relative expression of ERβ and GPR30 was normalized to ERα. Data are presented as mean ± SEM. N=3. ^*^P<0.05, ^**^P<0.01, ^***^P<0.001 and ^****^P<0.0001 were determined by one-way ANOVA.

The basal expression of ER subtypes (ERα, ERβ and GPR30) at mRNA and protein level in A431 cells was determined by RT-qPCR and Western blotting, respectively. As shown in Figure 1A(Fig. 1), among these three ER subtypes, the expression of GPR30 mRNA was the highest (P<0.001). ESR1 mRNA expression was higher than ESR2 (P<0.01). In order to further confirm these results, quantification of ERα, ERβ and GPR30 protein expression was carried out in A431 cells. As shown in Figure 1B(Fig. 1), the expression of GPR30 was still significantly the highest (P<0.0001), whereas the expression of ERβ was higher than ERα (P<0.05). These results suggested that GPR30 was the dominant ER subtype expressed in A431 cells, followed by ERβ and ERα.

### ER subtypes in A431 cells are localized in the cytoplasm

**Figure 2:**
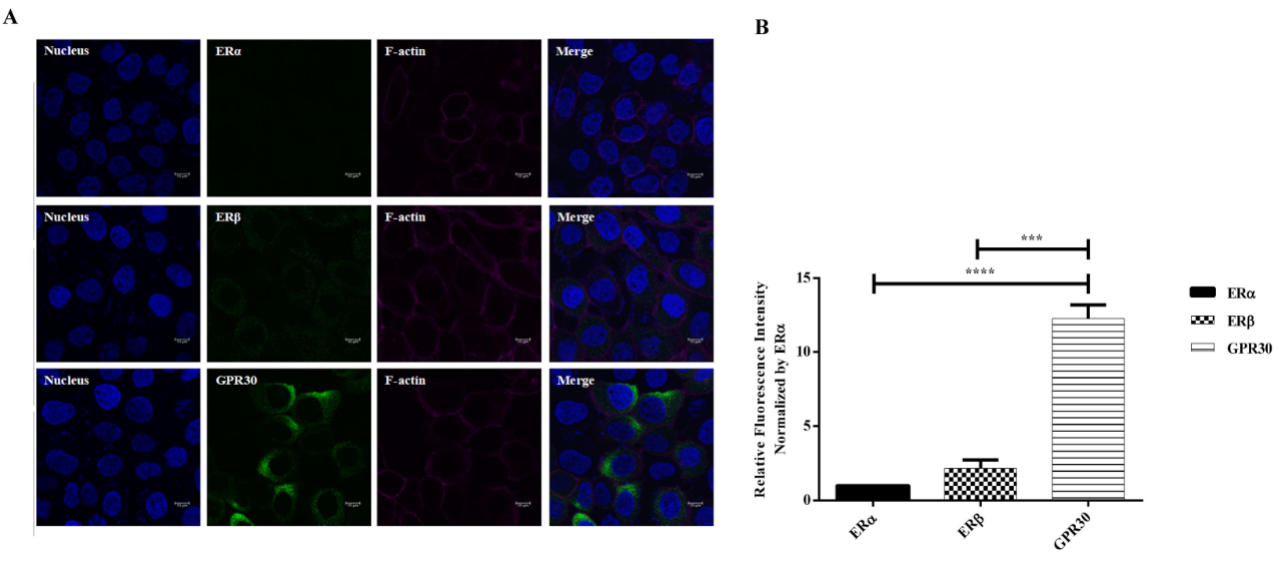
Determination of subcellular localization of ER subtypes in A431 cells using confocal microscopy. (A) Nucleus was stained with DAPI as blue, ER subtypes were stained with Alexa 488 as green and F-actin was stained with phalloidin as red. The merged images show the subcellular localization of ER subtypes in the cells. Magnification is 63x. (B) Relative fluorescence intensity of ER subtypes was measured relative to ERα. Image analysis was performed by Nikon NIS elements software. Data are presented as mean ± SEM. N=3. ^***^P<0.001 and ^****^P<0.0001 were determined by one-way ANOVA.

The localization of ER subtypes in A431 cells was visualized by confocal microscopy. As shown in Figure 2A(Fig. 2), all the 3 ER subtypes were localized in the cytoplasm. No nuclear staining of the ERs was observed. No green staining was observed in negative control groups (data not shown). The relative fluorescence intensity of ER subtype expression was consistent with our Western blotting results (Figure 2B(Fig. 2)). 

### Concentrations of ER agonists used do not affect the viability of A431 cells

**Figure 3:**
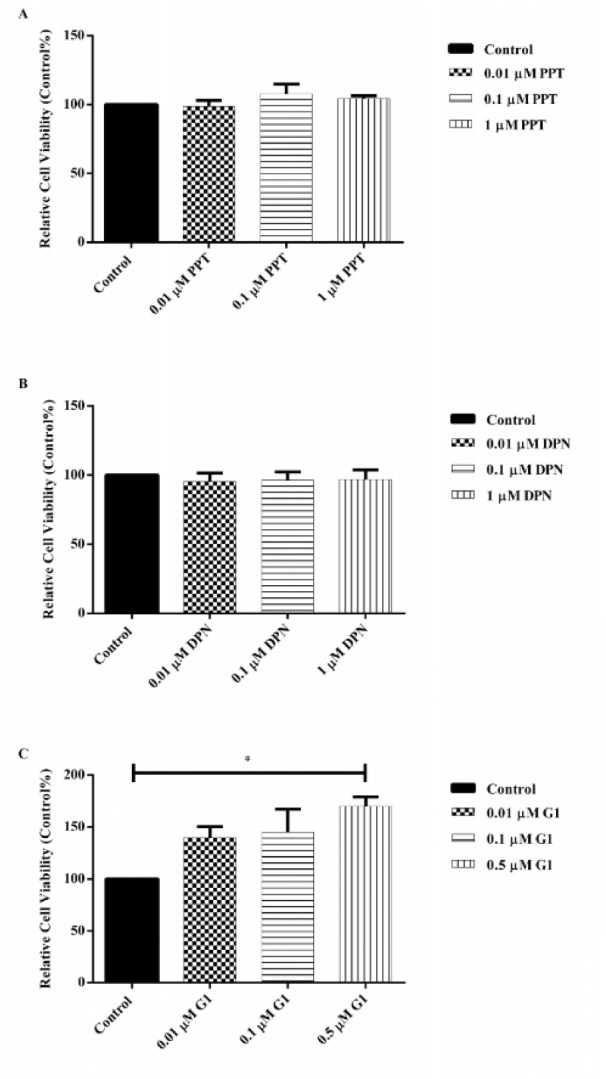
Cell cytotoxicity test for ER agonists on A431 cells. A431 cells were treated with PPT (A), DPN (B) or G1 (C) for 24 hours and cell viability was estimated using MTT assay. All the doses of drugs used in this study showed no cytotoxicity to cells. G1 increased cell viability in a dose-dependent manner. Data are presented as mean ± SEM. N=3-4. ^*^P<0.05 compared to control was determined by one-way ANOVA.

MTT assay was performed to check cell viability of A431 cells treated with various concentrations of ER agonists. When compared to vehicle treated cells, no significant differences in cell viability were detected in cells treated with various concentrations of drugs except for G1 (0.5 µM). These results indicated that the doses of the drugs used in this study were not cytotoxic to the cells (Figure 3(Fig. 3)). 

### ER agonists differentially modulate Cyclin D1 (CCND1) and CD55 mRNA expression in A431 cells

**Figure 4:**
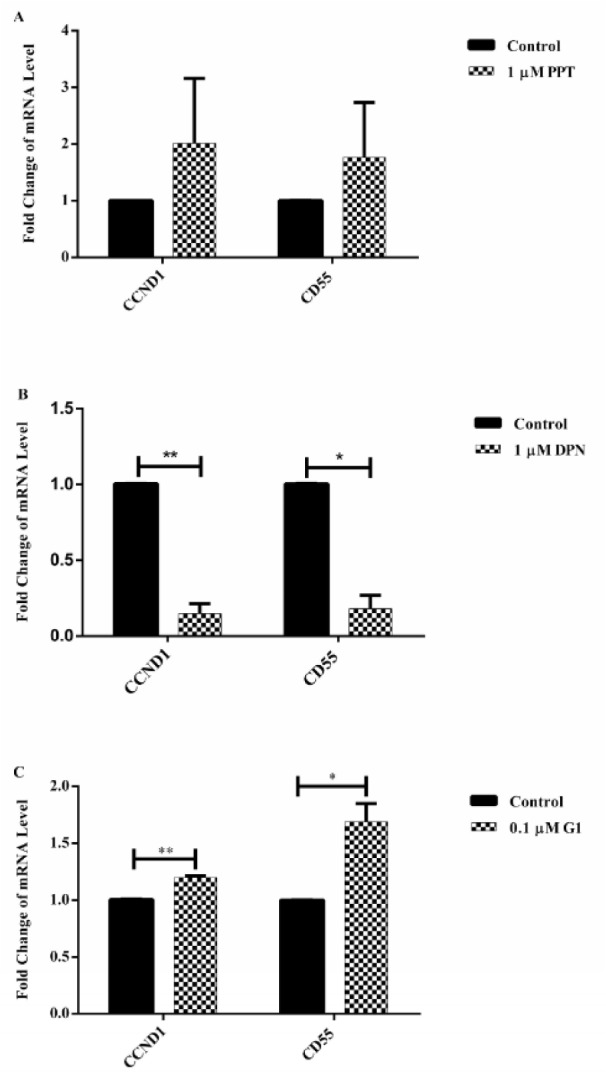
ER agonist modulation on CCND1 and CD55 mRNA expression in A431 cells. A431 cells were treated with PPT (A), DPN (B) or G1 (C) for 24 hours. Expression of CCND1 and CD55 mRNA was assessed by RT-qPCR. RPL13A was used as an internal control. mRNA expression of treatment group samples was normalized to the control sample. Data are presented as mean ± SEM. N=3. ^*^P<0.05 and ^**^P<0.01 compared to control were determined by unpaired t test.

The modulation of CCND1 and CD55 mRNA expression was determined in A431 cells in response to ER agonist based activation. Compared to control group, an increase in CCND1 and CD55 mRNA expression was observed in cells treated with PPT, however, this increase did not reach statistical significance (P>0.05, Figure 4A(Fig. 4)). A significant downregulation in CCND1 (P<0.01) and CD55 (P<0.05) mRNA expression in the cells treated with DPN was obtained (Figure 4B(Fig. 4)). A significant upregulation in CCND1 (P<0.01) and CD55 (P<0.05) mRNA expression in the cells treated with G1 was observed (Figure 4C(Fig. 4)). 

### ER agonists differentially modulate Cyclin D1 and CD55 protein expression in A431 cells

**Figure 5:**
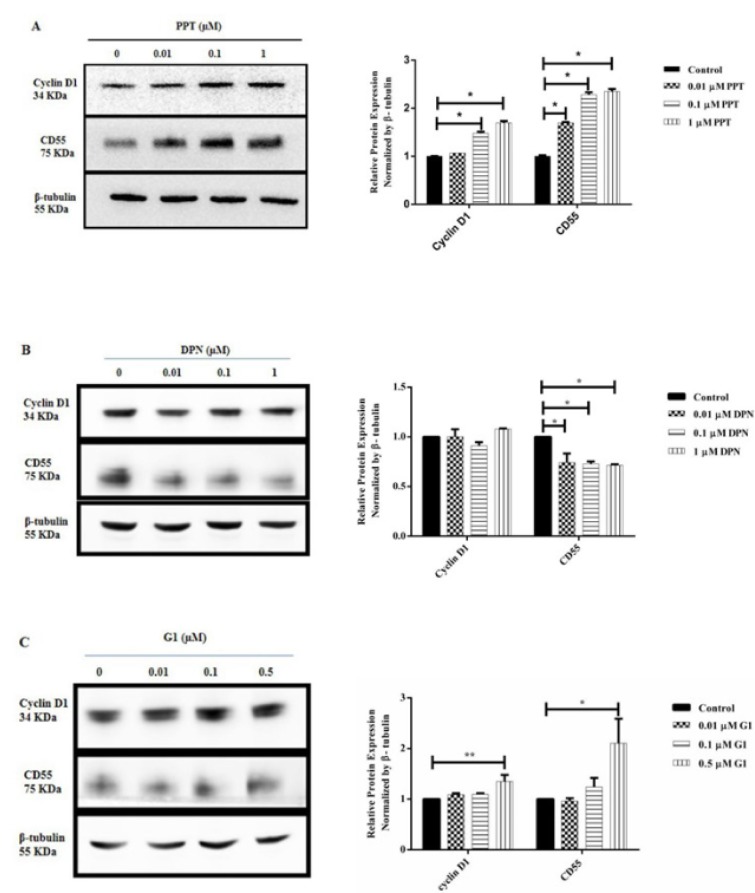
ER agonist modulation on Cyclin D1 and CD55 protein expression in A431 cells. A431 cells were treated with PPT (A), DPN (B) or G1 (C) for 24 hours. Expression of Cyclin D1 and CD55 protein was assessed by Western blotting assay. β-tubulin was used as an internal control. Protein expression of treatment group samples was normalized to the control sample. Data are presented as mean ± SEM. N=3-4. ^*^P<0.05 and ^**^P<0.01 compared to control were determined by one-way ANOVA.

The modulation of Cyclin D1 and CD55 protein expression was determined in A431 cells in response to different concentrations of ER agonists. Compared to control, Cyclin D1 and CD55 expression was significantly upregulated in the cells treated with PPT in dose-dependent manner (P<0.05, Figure 5A(Fig. 5)). Various concentrations of DPN treatment had comparable effects on Cyclin D1 protein expression; however, a dose-dependent manner of downregulation in CD55 protein expression was obtained (P<0.05, Figure 5B(Fig. 5)). Both Cyclin D1 (P<0.01) and CD55 (P<0.05) expression was significantly upregulated in cells treated with highest dose of G1 (0.5 µM) (Figure 5C(Fig. 5)). 

### GPR30 antagonist G15 downregulates Cyclin D1 and CD55 expression in A431 cells

**Figure 6:**
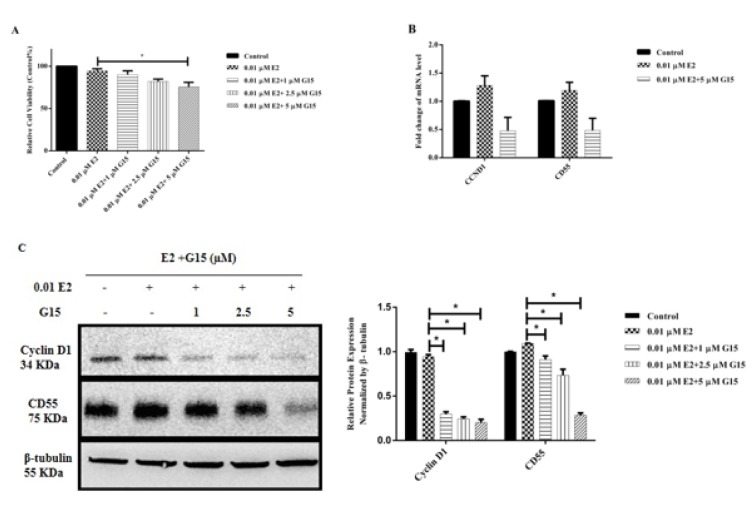
GPR30 antagonist G15 modulation on Cyclin D1 and CD55 expression in A431 cells. A431 cells were treated with 0.01 µM E2 with or without G15 for 24 hours. (A) Cell viability of cells treated with 0.01 µM E2+5 µM G15 was reduced compared to 0.01 µM E2 group. (B) Modulation of G15 on CCND1 and CD55 mRNA expression in A431 cells was assessed by RT-qPCR. RPL13A was used as an internal control. mRNA expression of treatment group samples was normalized to the control sample. (C) Modulation of G15 on Cyclin D1 and CD55 protein expression in A431 cells was assessed by Western blotting assay. β-tubulin was used as an internal control. Protein expression of treatment group samples was normalized to the control sample. Data are presented as mean ± SEM. N=3-4. ^*^P<0.05 compared to 0.01 µM E2 group was determined by one-way ANOVA.

In our study, GPR30 was found to be the dominant ER subtype expressed in A431 cells and GPR30 activation by G1 upregulated both Cyclin D1 and CD55 expression. In order to confirm the role of GPR30 in regulating Cyclin D1 and CD55 in A431 cells, GPR30 antagonist G15 treatment on cells was performed in presence of E2. MTT assay was performed to check the cell viability of A431 cells treated with various concentrations of E2 and G15. Compared to the E2 group, none of the drug doses used affected the cell viability, except significant decrease in the cells treated with E2+5 µM G15 (P<0.05, Figure 6A(Fig. 6)). However, the cell viability was above 75 %, even at the highest concentration of G15. A downregulation of CCND1 and CD55 mRNA expression was observed in the cells treated with E2+5 µM G15 compared to E2 group, however, not significant (P>0.05, Figure 6B(Fig. 6)). In addition, we observed a remarkable dose-dependent reduction of Cyclin D1 and CD55 protein expression in E2+G15 groups compared to E2 group (P<0.05, Figure 6C(Fig. 6)). 

## Discussion

Skin is recognized to be an estrogen responsive organ and various types of skin cells express ERs, such as keratinocytes (Pomari et al., 2015[30]). Despite the clinical evidence for hormonal etiology for melanoma and non-melanoma skin cancers (Driscoll and Grant-Kels, 2009[8]; Kuklinski et al., 2016[18]), we have currently a poor understanding of the role of estrogen and ERs in malignant transformation of various cutaneous cell types. The incidence of skin cancers is increasing particularly in Caucasians with over a million cases detected each year (Geller and Annas, 2003[11]). cSCC accounts for 20 % of all non-melanoma skin cancers and is the second common skin cancer after basal cell carcinoma (Eisemann et al., 2014[10]). Both animal and epidemiological studies strongly suggest gender based etiology for cSCC with male gender showing higher cSCC incidence (Thomas-Ahner et al., 2007[35]; Pollock, 2001[29]). The present study was conducted in human cSCC cell line to confirm the expression of all three ERs and to further investigate their activation by ER agonists on modulation of tumor markers Cyclin D1 and CD55. This study would further help to clear the current controversies that exist in the field regarding the hormonal based regulation and role of ERs in human cSCC.

Human cSCC development involves cellular malignant proliferation of cutaneous squamous epithelial cells. Normal human epidermal keratinocytes have been reported to express ERα, ERβ and GPR30 (Pomari et al., 2015[30]) with comparable basal expression. Mouse related cSCC cell lines and tissue studies have reported higher expression of ERα than ERβ and positive correlation between ratio of ERα/ERβ and malignancy (Mancuso et al., 2009[22]; Logotheti et al., 2012[21]). Another study observed low level of ERα expression in human SCC cells (Ku and Crowe, 2007[17]). However, none of these studies have investigated GPR30 expression. The present study is the first to report the expression of all three ER subtypes in cSCC A431 cells showing GPR30 expression being the highest followed by ERβ and ERα. To our knowledge, we are also the first to show the cytoplasmic localization of all three ERs in the cells. ERα and ERβ are classical nuclear ERs, however, we observed their expression in the cytoplasm only. GPR30 is known to be a membrane ER (Mangiamele et al., 2017[23]). In our study, GPR30 expression in A431 cells was detected in the cytoplasm, however, its membrane expression was not confirmed in this study.

Proliferation markers are frequently studied to track cancer prognosis. Cyclin D1 is a cell cycle regulatory protein during cell proliferation and is dysregulated more frequently than other proliferation markers in tumors (Qie and Diehl, 2016[31]). It is established that ERα and GPR30 positively correlate with Cyclin D1 expression in gastric (Tang et al., 2017[33]) and ovarian (Albanito et al., 2007[2]) cancers. A study has shown that GPR30 activation could induce the expression of Cyclin D1 in breast cancer MCF-7 cells (Lei et al., 2019[19]). Consistent with these previous studies, our results suggested that both PPT (ERα agonist) and G1 (GPR30 agonist) could induce Cyclin D1 protein expression in A431 cells. In various types of hormone dependent cancers, ERα and ERβ have been shown to differentially influence the progression of cancer (Thomas and Gustafsson, 2011[34]) by exerting opposite effects on cellular proliferation and apoptosis. A study in A431 cells has demonstrated that ERβ agonist, Erb-041, reduces Cyclin D1 protein expression (Chaudhary et al., 2014[5]). In our study, by using another type of ERβ agonist, DPN, a decrease of Cyclin D1 was observed at mRNA level, however, not at protein level. We attribute these differences in mRNA versus protein results due to the specific time point selection. In summary, our findings demonstrate that both ERα and GPR30 activation are able to induce Cyclin D1 expression, thus may be involved in cSCC cell malignant progression. In contrast, ERβ activation was able to suppress Cyclin D1 expression, suggesting its protective role in inhibiting cSCC cell malignant progression.

CD55 has been detected in various cancers (Whitlow and Klein, 1997[38]; Cheung et al., 1988[7]) with 4-100 fold higher expression than normal cells (Li et al., 2001[20]). CD55 expression can be regulated by estrogen (Nowicki and Nowicki, 2013[27]), but the exact action of estrogen or ER subtype activation on CD55 expression in cSCC cells remains unknown. This study is the first to investigate ER activation on CD55 expression in human cSCC cells. Our results demonstrate that DPN can reduce CD55 expression at both mRNA and protein level in A431 cells, implicating its use in activation of complement system to kill cancer cells. In contrast, PPT and G1 were able to induce CD55 expression at protein level in A431 cells, implying that ERα and GPR30 activation may help cells to escape the complement attack and drive cell malignant progression. Taken together, our results suggest that ERα, ERβ and GPR30 activation exert different roles in cSCC cell malignant progression via regulating CD55.

In our study, GPR30 was identified as the dominant ER subtype in A431 cells and its activation induced both Cyclin D1 and CD55 expression, suggesting its role in driving cSCC cell malignant progression. GPR30 modulation on Cyclin D1 and CD55 expression was further confirmed by treating A431 cells with GPR30 antagonist, G15, which resulted in remarkable decrease in Cyclin D1 and CD55 protein expression. A recent study has shown that G15 is able to suppress oral SCC cell growth, thus supporting our results (Bai et al., 2013[3]).

In summary, this study demonstrates that tumor markers Cyclin D1 and CD55 in A431 cells can be differentially modulated via activation of three ER subtypes, suggesting the involvement of ER signals in cancer cell progression. These results may have important clinical significance related to cSCC pathogenesis. Currently, there are no biomarkers for predicting malignant behavior of certain subtypes of cSCC. ER subtype and their relative expression on cells may serve as novel malignant stage-specific biomarker to predict worst prognosis in cSCC. Finally, our results suggest that ERβ activation or blocking of GPR30 activation may serve as a novel therapeutics for treating human cSCC.

## Acknowledgements

We thank Dr. Subhas Das and Michael Anderson (OSU-CHS) for their expert advice in molecular biology and confocal microscopy. We also would like to thank Ayantika Sen and Sarah Groover (OSU-CHS) for providing critique of this manuscript.

## Interest statement

The authors declare that they have no conflict of interest.

## Funding

This project was supported by Cancer Sucks Inc. (Bixby, Oklahoma) research grant to Dr. Rashmi Kaul.

## Figures and Tables

**Table 1:**
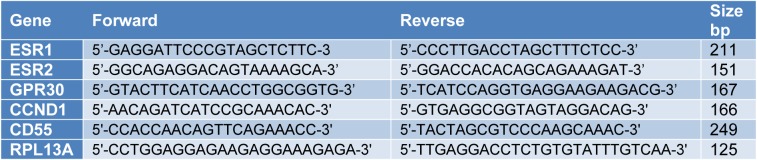
Primers used in RT-PCR

## References

[1] Ahlin C, Lundgren C, Embretsen-Varro E, Jirstrom K, Blomqvist C, Fjallskog M (2017). High expression of cyclin D1 is associated to high proliferation rate and increased risk of mortality in women with ER-positive but not in ER-negative breast cancers. Breast Cancer Res Treat.

[2] Albanito L, Madeo A, Lappano R, Vivacqua A, Rago V, Carpino A (2007). G protein-coupled receptor 30 (GPR30) mediates gene expression changes and growth response to 17beta-estradiol and selective GPR30 ligand G-1 in ovarian cancer cells. Cancer Res.

[3] Bai LY, Weng JR, Hu JL, Wang D, Sargeant AM, Chiu CF (2013). G15, a GPR30 antagonist, induces apoptosis and autophagy in human oral squamous carcinoma cells. Chem Biol Interact.

[4] Burton KA, Ashack KA, Khachemoune A (2016). Cutaneous squamous cell carcinoma: a review of high-risk and metastatic disease. Am J Clin Dermatol.

[5] Chaudhary SC, Singh T, Talwelkar SS, Srivastava RK, Arumugam A, Weng Z (2014). Erb-041, an estrogen receptor-beta agonist, inhibits skin photocarcinogenesis in SKH-1 hairless mice by downregulating the WNT signaling pathway. Cancer Prev Res (Phila).

[6] Chen HH, Chen SP, Zheng QL, Nie SP, Li WJ, Hu XJ (2018). Genistein promotes proliferation of human cervical cancer cells through estrogen receptor-mediated PI3K/Akt-NF-kappaB pathway. J Cancer.

[7] Cheung NK, Walter EI, Smith-Mensah WH, Ratnoff WD, Tykocinski ML, Medof ME (1988). Decay-accelerating factor protects human tumor cells from complement-mediated cytotoxicity in vitro. J Clin Invest.

[8] Driscoll MS, Grant-Kels JM (2009). Estrogen receptor expression in cutaneous melanoma. Arch Dermatol.

[9] Durrant LG, Chapman MA, Buckley DJ, Spendlove I, Robins RA, Armitage NC (2003). Enhanced expression of the complement regulatory protein CD55 predicts a poor prognosis in colorectal cancer patients. Cancer Immunol Immunother.

[10] Eisemann N, Waldmann A, Geller AC, Weinstock MA, Volkmer B, Greinert R (2014). Non-melanoma skin cancer incidence and impact of skin cancer screening on incidence. J Invest Dermatol.

[11] Geller AC, Annas GD (2003). Epidemiology of melanoma and nonmelanoma skin cancer. Semin Oncol Nurs.

[12] Guo L, Yilamu D, Sun L, Liu S, Ma F (2015). Association among the expression of beta-catenin, cyclin D1 and estrogen receptor-beta in human breast cancer. Exp Ther Med.

[13] Heldring N, Pike A, Andersson S, Matthews J, Cheng G, Hartman J (2007). Estrogen receptors: how do they signal and what are their targets. Physiol Rev.

[14] Huang W, Chen Y, Liu Y, Zhang Q, Yu Z, Mou L (2015). Roles of ERbeta and GPR30 in proliferative response of human bladder cancer cell to estrogen. Biomed Res Int.

[15] Ito T, Tachibana M, Yamamoto S, Nakashima J, Murai M (2001). Expression of estrogen receptor (ER-alpha and ER-beta) mRNA in human prostate cancer. Eur Urol.

[16] Kawano M (2000). Complement regulatory proteins and autoimmunity. Arch Immunol Ther Exp (Warsz).

[17] Ku TK, Crowe DL (2007). Coactivator-mediated estrogen response in human squamous cell carcinoma lines. J Endocrinol.

[18] Kuklinski LF, Zens MS, Perry AE, Gossai A, Nelson HH, Karagas MR (2016). Sex hormones and the risk of keratinocyte cancers among women in the United States: A population-based case-control study. Int J Cancer.

[19] Lei B, Sun S, Zhang X, Feng C, Xu J, Wen Y (2019). Bisphenol AF exerts estrogenic activity in MCF-7cells through activation of Erk and PI3K/Akt signals via GPER signaling pathway. Chemosphere.

[20] Li L, Spendlove I, Morgan J, Durrant LG (2001). CD55 is over-expressed in the tumour environment. Br J Cancer.

[21] Logotheti S, Papaevangeliou D, Michalopoulos I, Sideridou M, Tsimaratou K, Christodoulou I (2012). Progression of mouse skin carcinogenesis is associated with increased ERalpha levels and is repressed by a dominant negative form of ERalpha. PLoS One.

[22] Mancuso M, Gallo D, Leonardi S, Pierdomenico M, Pasquali E, De Stefano I (2009). Modulation of basal and squamous cell carcinoma by endogenous estrogen in mouse models of skin cancer. Carcinogenesis.

[23] Mangiamele LA, Gomez JR, Curtis NJ, Thompson RR (2017). GPER/GPR30, a membrane estrogen receptor, is expressed in the brain and retina of a social fish (Carassius auratus) and colocalizes with isotocin. J Comp Neurol.

[24] Multhoff G, Molls M, Radons J (2011). Chronic inflammation in cancer development. Front Immunol.

[25] Murray KP, Mathure S, Kaul R, Khan S, Carson LF, Twiggs LB (2000). Expression of complement regulatory proteins-CD 35, CD 46, CD 55, and CD 59-in benign and malignant endometrial tissue. Gynecol Oncol.

[26] Nakamura Y, Felizola SJ, Kurotaki Y, Fujishima F, McNamara KM, Suzuki T (2013). Cyclin D1 (CCND1) expression is involved in estrogen receptor beta (ERbeta) in human prostate cancer. Prostate.

[27] Nowicki B, Nowicki S (2013). DAF as a therapeutic target for steroid hormones: implications for host-pathogen interactions. Adv Exp Med Biol.

[28] Nugent EK, Brooks RA, Barr CD, Case AS, Mutch DG, Massad LS (2011). Clinical and pathologic features of vulvar intraepithelial neoplasia in premenopausal and postmenopausal women. J Low Genit Tract Dis.

[29] Pollock J (2001). Cutaneous squamous-cell carcinoma. N Engl J Med.

[30] Pomari E, Dalla Valle L, Pertile P, Colombo L, Thornton MJ (2015). Intracrine sex steroid synthesis and signaling in human epidermal keratinocytes and dermal fibroblasts. FASEB J.

[31] Qie S, Diehl JA (2016). Cyclin D1, cancer progression, and opportunities in cancer treatment. J Mol Med (Berl).

[32] Sun M, Xie HF, Tang Y, Lin SQ, Li JM, Sun SN (2017). G protein-coupled estrogen receptor enhances melanogenesis via cAMP-protein kinase (PKA) by upregulating microphthalmia-related transcription factor-tyrosinase in melanoma. J Steroid Biochem Mol Biol.

[33] Tang W, Liu R, Yan Y, Pan X, Wang M, Han X (2017). Expression of estrogen receptors and androgen receptor and their clinical significance in gastric cancer. Oncotarget.

[34] Thomas C, Gustafsson JA (2011). The different roles of ER subtypes in cancer biology and therapy. Nat Rev Cancer.

[35] Thomas-Ahner JM, Wulff BC, Tober KL, Kusewitt DF, Riggenbach JA, Oberyszyn TM (2007). Gender differences in UVB-induced skin carcinogenesis, inflammation, and DNA damage. Cancer Res.

[36] Tian J, Wang Y, Zhang X, Ren Q, Li R, Huang Y (2017). Correction to: Calycosin inhibits the in vitro and in vivo growth of breast cancer cells through WDR7-7-GPR30 Signaling. J Exp Clin Cancer Res.

[37] van Diest PJ, Brugal G, Baak JP (1998). Proliferation markers in tumours: interpretation and clinical value. J Clin Pathol.

[38] Whitlow MB, Klein LM (1997). Response of SCC-12F, a human squamous cell carcinoma cell line, to complement attack. J Invest Dermatol.

[39] Yang CC, Chu KC, Chen HY, Chen WC (2002). Expression of p16 and cyclin D1 in bladder cancer and correlation in cancer progression. Urol Int.

[40] Yesantharao P, Wang W, Ioannidis NM, Demehri S, Whittemore AS, Asgari MM (2017). Cutaneous squamous cell cancer (cSCC) risk and the human leukocyte antigen (HLA) system. Hum Immunol.

[41] Yue W, Wang JP, Li Y, Fan P, Liu G, Zhang N (2010). Effects of estrogen on breast cancer development: Role of estrogen receptor independent mechanisms. Int J Cancer.

[42] Zhu CX, Xiong W, Wang ML, Yang J, Shi HJ, Chen HQ (2018). Nuclear G protein-coupled oestrogen receptor (GPR30) predicts poor survival in patients with ovarian cancer. J Int Med Res.

